# Russian nursing students’ knowledge level and attitudes in the context of human immunodeficiency virus (HIV) – a descriptive study

**DOI:** 10.1186/s12912-014-0053-7

**Published:** 2015-01-08

**Authors:** Tarja Suominen, Laura Laakkonen, Dmitry Lioznov, Maya Polukova, Svetlana Nikolaenko, Liudmila Lipiäinen, Maritta Välimäki, Jari Kylmä

**Affiliations:** School of Health Sciences, Nursing Science, University of Tampere, Tampere, Finland; Lahti Polytechnic, Lahti, Finland; Center for Preventive Medicine, Pavlov State Medical University, 6/8 Lev Tolstoy St., 197089 St. Petersburg, Russia; Department of Higher Nursing Education, Pavlov State Medical University, 6/8 Lev Tolstoy St., 197089 St. Petersburg, Russia; Center for Chronic Viral Infections Research, Pavlov State Medical University, 6/8 Lev Tolstoy St., 197089 St. Petersburg, Russia; School of Health Sciences, University of Tampere, Tampere, Finland; Department of Nursing Science and Turku University Hospital, University of Turku, Turku, Finland

**Keywords:** Knowledge, Attitudes, Care, HIV, PLWHA, Nursing student, Russia

## Abstract

**Background:**

The purpose of this study was to describe the knowledge of Russian nursing students regarding HIV and Acquired Immuno-Deficiency Syndrome (AIDS), and their attitudes towards caring for people/patients living with HIV or AIDS (PLWHA - People Living With HIV/AIDS) and their possible homophobic attitudes. The HIV epidemic in Russia is substantial and increasing rapidly. Hence this study provides important new information regarding this phenomenon.

**Methods:**

The data was collected by questionnaire from students in three nursing schools (*n* = 102, response rate 95.3%). The data was analyzed using PASW Statistics version 18. For computing the level of the students’ AIDS knowledge, all correct answers were recorded as equal to (1), while all incorrect and “Don’t know” answers were recorded as equal to (0). Each respondent’s scores were totaled and individual scores were analyzed using regression analysis. The effect of demographic variables on the average scores of attitudes was also subjected to regression analysis.

**Results:**

Overall, students’ knowledge level regarding HIV and AIDS was moderate (range 5–26). Of a maximum score of 33, the mean of correct answers was 19.8 (*SD* = 3.70). Nursing students’ attitudes were quite negative and they also demonstrated homophobic attitudes. The mean scale score for nursing students’ general attitude was 2.75, and for homophobic attitudes it was 3.3 (*min* = 1, *max* = 5). Only the background factor of gender correlated with the homophobic level demonstrated (*p* = .05, *β* = −.67). Nursing students’ overall willingness to provide care for PLWHA was associated with their attitudes (*p* = .003, *β* = −.534).

**Conclusions:**

Given that the HIV epidemic in Russia is both substantial and increasing, it is essential to improve HIV nursing education to provide sufficient and up-to-date information about HIV and also to prepare nursing students for caring for PLWHA. In doing so, this may help to address both the deficits in student knowledge, and also modify their attitude towards PLWHA.

## Background

Globally, new HIV infections have dropped by 38% since 2001, but there were 2.1 million people newly infected in 2013, and an estimated 22 million people who are not accessing life-saving treatment [1]. Furthermore, there are some countries where the HIV epidemic is growing (such as in Eastern Europe and Central Asia) where the number of people living with HIV has almost tripled (1.4 million people living with HIV in year 2009). In Russia and Ukraine, the prevalence of HIV is one percent or higher. In these countries, the most common routes of transmission are injection drug use (IDU) and sexual contact [1]. High HIV incidence among IDU attests to continued growth of the epidemic [2,3].

In present day Russia it is important to emphasize the activities in HIV prevention, treatment, care, and support [1]. Antiretroviral Therapy (ARVT) is available for everybody who needs it, and there are national recommendations for choosing ARVT drugs for different populations and situations.

The Russian Federation has conducted some sporadic research on the stigma of chronic viral infections. In 2008, the Russian Public Opinion Research Center (WCIOM) conducted surveys on the awareness of Russians about HIV infection, and the relation this had to HIV-infected people (3200 residents surveyed in 140 settlements of Russia). It turned out that if a family member became infected, 19% of respondents would change their attitude toward them for the worst. In the case of a housemate or a colleague at work, the figure raised to 23% and 28% respectively [4]. At the same time, 4% of respondents would limit their communication with infected relatives to a minimum, 14% would do so with a colleague and 23% with a neighbor. Most respondents would express strong concern if their child were in a group of HIV-infected children (62%), or if the child were under the supervision of an HIV-positive nurse (73%) [5]. In Russia, the mental constructs of people relating to HIV/AIDS may differ from formal observations and epidemiological data. It should be mentioned that homosexuality is stigmatized in Russia and we do not know the real data regarding HIV in the homosexual population. Previous research has shown that in nursing schools, students know of IDU as being a main route for acquiring HIV but often believe that all homosexuals have HIV because it is a risk-laden way for potential HIV acquisition. There are guidelines regarding Universal Precautions and available personal protection equipment, but nurses are taught ethical standards that support the rights of all patients to receive essential services in health care. This therefore raises a disparity between taught knowledge and personal attitudes.

Medical and nursing staff are the most important groups in the prevention of HIV. Therefore, medical and nursing education must include updated information on HIV and AIDS-related issues. The knowledge and attitudes of students towards caring for people with HIV or AIDS (PLWHA) is thus of vital importance, since they will develop into future health professionals. Additionally, previous studies (e.g. [6]) have shown that increased HIV and AIDS-related education has reduced negative attitudes toward PLWHA.

### Literature review

Internationally, nursing students’ knowledge levels regarding HIV and AIDS-related issues have been inconsistent and differences have been noticed in various areas related to HIV and AIDS. Whilst their knowledge level has been reported to be good in European countries such as Finland, the UK, Turkey and Germany [7-11], gaps and weaknesses have also been identified in the same countries (e.g. [7,10-14]). Quite poor knowledge levels and large knowledge gaps have however been reported in Asia and Africa, in countries such as Nepal and Ghana (e.g. [13,15-17]).

The associations between background factors and knowledge levels in the field have been previously identified. For example, male students have been found to be more knowledgeable than female students [7]. Age has been found to be positively correlated with knowledge level among nursing students [7,10]. Some studies suggest that single students have a higher knowledge level than those who are married or widowed/divorced [8,18]. Contrastingly however, some studies have found that married and cohabiting students have a higher knowledge level [10]. If students had cared for a patient with HIV [8,10] or knew someone with AIDS, then they were also likely to prove more knowledgeable [8]. Also, students with less work experience tended to have a higher knowledge level of HIV and AIDS, as did those with a higher level of education [10].

Apart from knowledge level, concerns have been expressed about nursing staff’s negative attitudes and care towards PLWHA [19,20]. Nursing students’ attitudes toward PLWHA have been studied in different countries, and attitudes have varied from rather positive [8,9,11], to average [7,21], to negative [12,13,16,17]. Nursing students have been reported to be apprehensive about caring for PLWHA [8,22] and associations between background factors and attitudes have been identified. Earlier experiences of caring for PLWHA or other contact with them appears to have a positive influence on attitude. A willingness to care for PLWHA has also been positively associated [9,18].

Homophobic attitudes vary across different countries, and may be linked with PLWHA. This was found to be the case in two studies: students with homophobic attitudes were less willing to care for persons infected with HIV and those with AIDS [9,14]. Earl and Penny [12] found high levels of homophobia among nursing students in the USA, however, some studies suggest that nursing students may in fact have less homophobic attitudes and/or rather positive attitudes [8,9,21]. In a study by Peate et al. [9], older nursing students and students with more work experience showed a more negative attitude toward homosexual patients. Those nursing students who had previous experience with PLWHA, had previously been asked to care or had already cared for PLWHA showed lower degrees of homophobic attitudes. Students who were more willing to care for PLWHA also showed more positive attitudes toward homosexual patients. Also, students with children showed more positive attitudes toward homosexual patients than those who did not [21]. It has also been seen that students with lower knowledge levels of HIV or AIDS, tended to express more homophobic attitudes than those who had a higher degree of knowledge [8,9].

A review of research studies concerning the attitudes of nursing students towards caring for PLWHA and their influencing factors was carried out, and identified the following themes: education and knowledge of HIV and AIDS; fear of contracting HIV; reluctance to care for people with HIV or AIDS; homophobia; stigma associated with HIV and AIDS. It also reported that there is a reluctance on the part of some nursing students in specific regions of the world to provide care for PLWHA [6], however the focus of this article is on Russia.

In Eastern Europe, the number of people with HIV has almost tripled since 2000 [1]. Nurses and nursing students increasingly care for people infected with HIV, people with AIDS and people who are at risk of contracting HIV. Nurses should therefore be knowledgeable about HIV infection and AIDS in order to be able to provide proper care for PLWHA.

### The nursing program in Russia

In Russia, nursing education is provided by way of a 3-year course. The qualification awarded is that of Qualified Nurse. The nursing curriculum includes an infectious diseases module where HIV infection and related issues are incorporated into the training program.

There has been little research on Russian nurses. Neither has there been much research on Russian nursing students, and especially regarding their knowledge and attitudes towards PLWHA in Russia. Hence this study provides important new information in this area of study.

### Purpose and research questions

The purpose of this study was to describe nursing students’ knowledge level regarding HIV and AIDS, and their attitudes toward PLWHA. The ultimate goal was to compile a knowledge base reflecting the present situation, in order to develop this aspect of nursing education.

The research questions were as follows:

1. What is the level of HIV and AIDS related knowledge among nursing students?
2. What associations can be identified between nursing students’ background factors and their level of knowledge regarding HIV and AIDS?
3. What are nursing students’ attitudes towards PLWHA and homosexual people?
4. What associations can be identified between nursing students’ background factors and attitudes towards PLWHA and homosexual people?

## Methods

### Data collection

A purposive sample was drawn from 3 out of 10 nursing schools in one metropolitan city in Russia, in a geographical area where the prevalence of HIV and AIDS is recorded as high; e.g. the HIV prevalence among IDUs has been reported to be as high as 50% (2). The data was collected between May and April 2010 after receiving permissions from all of the study institutions according to their research standards. Graduating nursing students completed the questionnaires during their pre-practical training period. After completing the questionnaire (about one hour), the students returned the completed questionnaires to the research assistant. The questionnaires were offered to 107 students and 102 students participated (response rate 95.3%). All of the students were due to graduate after 3 or 4 years of education, depending on their previous basic school level.

### Instrument

The data was collected using a questionnaire originally developed in the USA [23], and modified for use in Europe by Finnish nursing scholars. The modified version has been used in Finland, the UK, Germany, Estonia, and Lithuania. The instrument has been translated by translation-back translation techniques [24] into Finnish, German, Estonian, Russian, and Lithuanian. The original instrument developers and medical experts have checked the continued validity of the updated instrument where for example, the term HIV was used instead of AIDS.

The questionnaire consisted of three sections: a) background questions, b) knowledge questions, and c) attitude questions. The background questions consisted of 14 demographic and other background items, including age, gender, marital status, children, time spent in nursing education, knowing a family member or anyone else who had HIV or AIDS, whether the informant had ever been asked to care for a person with HIV or AIDS, had ever refused to care for a person with HIV or AIDS, and whether they would be willing to care for a person with HIV or AIDS.

The next section consisted of a knowledge test, with 33 questions about the nursing student’s knowledge of HIV and AIDS. This included questions about HIV and AIDS immunology, modes of transmission, disease etiology, risk groups and universal precautions. Possible responses were 1 = true, 2 = false, 3 = don’t know.

Lastly, 35 items focused on nursing students’ attitudes towards PLWHA and the disease itself. The attitude scale had two subscales: general attitudes and homophobic attitudes. The general attitude subscale had 26 items evaluating nursing students’ attitudes toward groups such as intravenous drug users, prostitutes etc., and items concerning their fears about personal safety, hopelessness, and their preparedness to meet the needs of persons infected with HIV. The homophobia subscale had nine items measuring nursing students’ attitudes toward homosexually-oriented people. Responses were given on a 5-point Likert scale (1 = strongly agree, 2 = agree, 3 = undecided, 4 = disagree and 5 = strongly disagree) – a score of 5 indicating the most positive attitude.

The instrument has been found to be reliable and valid. In previous studies, Cronbach’s alpha values for the attitude scales have ranged between 0.86 and 0.95 [21]. In this study the Cronbach’s alpha values were found to be 0.87 for general attitudes and 0.92 for homophobia.

### Ethical considerations

Permissions to conduct the study were received based on the protocols of the educational institutions concerned. As no patients were involved, a statement from the ethical committees was not needed. Students gave their oral consent, which was further implied by their voluntary completion and return of the questionnaire. Students’ anonymity was guaranteed.

### Data analysis

The data was analyzed using PASW Statistics version 18. The demographic variables were described using descriptive statistics. For computing the level of the students’ AIDS-related knowledge, all correct answers were recorded as being equal to (1), while all incorrect and “Don’t know” answers were recorded as equal to (0). Each respondent’s scores were totaled and individual scores were analyzed using regression analysis.

Nursing students’ general and homophobic attitudes were described using descriptive statistics. To calculate the average scales of attitudes, responses from each respondent were totaled and divided by the amount of responses. The effect of demographic variables on the average scores of general and homophobic attitudes was analyzed using regression analysis. Only statistically significant results (p < 0.05) have been reported in results.

## Results

### Participants

Of the nursing students who completed the questionnaire (n = 102), 16% were 18 years of age, 36% were 19, 33% were 20 and 16% were 21 years of age or older. The majority (85%) were single, female (89%), and 9% had children. A great majority (88%) spoke Russian as their native language. 70% were Orthodox Christians, and for 54% of all respondents, their religious faith was seen as important.

Sixteen percent of the students reported having known a family member, friend or other person with HIV or AIDS. The proportion of students who had been asked to care for PLWHA was 20%. 18% reported that they have cared for PLWHA. 7% had refused care for PLWHA. Overall however, 80% of the students were not generally willing to care for PLWHA.

### Nursing students’ knowledge level

The mean of the correct answers for the 33 item knowledge test was 19.8 points (*min* = 5, *max* = 26, *SD* = 3.704). In the following results, we present at item level, some results which are thought to be among the most relevant for a nursing student to know when entering nursing practice.

The study showed that students’ knowledge of transmission-related issues varied widely (Table 1). About 60% of students knew that HIV is not found in large amounts in saliva, tears, and urine. Almost 70% of the students knew that HIV cannot be transmitted by casual contact. 62% of the students knew that condoms do not give 100% protection against contracting HIV through sexual practices, 15% of the students reported the opposite. Somewhat shockingly, 11% of the nursing students thought that all homosexuals had HIV (whilst 72% chose the correct answer, a further 17% did not know).

**Table 1:** **Percentages of nursing students’ knowledge of HIV/AIDS (** ***main selected issues*** **)**

	***True %***	***False %***	***Don’t know %***
AIDS is a disease that attacks the human immune system (n = 102).	96	2	2
There is no cure for AIDS (n = 100).	50	28	22
Individuals at high risk for contracting HIV include intravenous drug abusers, those who practice unprotected sex and prostitutes (n = 102).	98	0	2
ARC is a stage of the disease caused by HIV where the HIV antibody test is positive and the person displays specific symptoms related to the stage (n = 100).	86	1	13
T-lymphocytes decrease when a person has AIDS (n = 102).	82	1	17
AIDS is a lethal disease (n = 101).	85	9	6
Symptoms of HIV occur within six months following infection with HIV (n = 102)	71	5	25
People could get infected by sharing needles with a HIV positive intravenous drug user (n = 102).	95	3	2
The risk of contracting HIV increases as the number of one’s sexual partners increases (n = 101).	84	8	8
Individuals can infect others with HIV without being ill themselves (n = 102).	85	6	9
Infections (opportunistic) are common complications of AIDS (n = 101).	46	7	48
Kaposi sarcoma is the most common form of cancer among homosexual men (n = 101).	45	13	43
HIV wasting syndrome is one of the causes of death among people with AIDS in developing countries (n = 100).	37	19	44
AIDS is rather thought of as a disease related to sexual risk behavior than as one related to sexual preferences (n = 101).	83	4	13
HIV can be transmitted by blood and blood products (n = 101).	96	2	2
Sexual transmission of HIV can occur in both homosexuals and heterosexual relationships (n = 101).	93	1	6
Consistent use of condoms may reduce transmission of HIV (n = 101).	86	2	12
The need for isolation required for patients hospitalized with HIV or AIDS depends on the specific infection present (n = 101).	65	10	25
Blood precautions should be used for individuals hospitalized for treatment of HIV or AIDS (n = 101).	98	0	2
Eye protection should be worn by all individuals in situations where blood and body fluids can be splashed into the eyes of others (n = 101).	93	3	4

### Percentages of nursing students’ knowledge of HIV/AIDS

The students’ knowledge of HIV-related precautions in hospital could have been better. 45% correctly identified that there is no good evidence that HIV cannot be transmitted to a fetus before birth. Only 36% of the students knew that a mask should not be worn routinely when dealing with individuals with HIV or AIDS, and only 18% knew that it is not necessary to wear complete protective equipment (i.e. a mask, eyewear, cap, gown and gloves) while dealing with people with HIV or AIDS. Only 14% of the students identified the statement that isolation of PLWHA in a sterile environment is an efficient way to prevent infection complications was incorrect.

None of the background factors collated in the study correlated with the nursing students’ knowledge level.

### Nursing students’ general attitudes

The mean score for the nursing students’ general attitude scale was 2.75 (*min* = 1.0 and *max* = 4.5, *SD* = 0.6), measured on a 5-point Likert scale. The majority of students were afraid of being in contact with a person with HIV or AIDS, and 62% would be concerned about the possibility of contracting HIV. About three-quarters of the students were worried about their family, friends or colleagues after they had come into contact with a person with HIV, although almost half of the students thought that dealing with people with HIV would not affect their relationships with others.

Slightly less than 40% of the students reported that they would not want to be assigned to care for PLWHA as they felt they were not competent to meet their intense physical and psychological needs. Almost 40% of the students felt that caring for a person who is dying was uncomfortable, whilst 75% felt that it was worthwhile to care for a PLWHA who is dying. Almost half of the students thought that they had received enough information to deal with people with HIV.

There were some differences in students’ attitudes towards particular groups with HIV or AIDS. Almost 90% of the students felt more sympathy towards people who had acquired HIV through blood transfusion, rather than through the use of IV drugs. Also, 60% of the students felt that they had little sympathy toward drug users with HIV, and a similar proportion felt more sympathetic towards those who had acquired the disease through blood transfusion rather than through sexual contact. However, 17% indicated that they would feel uncomfortable dealing with PLWHA if the patient was a child; 22% if the person was a hemophiliac; 35% if the person was bisexual; 60% if the person was an IV drug user; and 64% if the person was a prostitute. 81% of students reported that those who care for PLWHA should receive additional payment, and 66% also stated that caring for PLWHA should be voluntary (Table 2).

**Table 2:** **Percentages of nursing students’ attitudes toward the care of PLWHA**

	***Agree %***	***Undecided %***	***Disagree %***
I should have the right to refuse to deal with persons with HIV or AIDS (n = 100).	78	15	7
I would refuse to care for a person with HIV or AIDS (n = 101).	41	36	24
Health care institutions should have the right to refuse to provide care to patients with HIV or AIDS (n = 101).	15	11	74
Providing care for people with HIV or AIDS should always be voluntary (n = 100).	66	14	20
People with HIV or AIDS should be cared for in a separate unit with specifically trained personnel (n = 101).	86	2	12
Those who care for people with HIV or AIDS should receive additional pay (n = 101).	81	14	5

### Percentages of nursing students’ attitudes toward the care of PLWHA

A statistically significant association was found between the nursing students’ overall willingness to provide care for PLWHA, and their general attitude score (*p* = .003, *β* = −.534).

### Nursing students’ homophobia

The mean score for the nursing students’ homophobia was 3.3 (*min* = 1.0, *max* = 5.0, *SD* = 1.08), measured on a 5-point Likert scale. A little under half of the students felt uncomfortable dealing with a homosexual with HIV or AIDS (43%), and half felt that they were not worried about dealing with homosexually-oriented persons. About 25% of the students felt that they did not feel comfortable dealing with gay females as they are with heterosexual women. The same proportion of the students felt that homosexuality is a lifestyle that should be condemned (agree = 25%, undecided = 34%, disagree = 42%). Almost 70% disagreed with the statement that homosexuals with HIV are getting what they deserve. 8% of the students thought that the same levels of respect and information should not be given to the partner of a homosexual, as would be given to the partner of a heterosexual (agree = 8%, undecided = 43%, disagree = 50%). Only 22% of the students felt comfortable being in contact with a homosexual person, as they would with a heterosexual. Almost 30% felt that their attitude toward homosexuals had become more negative since the beginning of the HIV and AIDS crisis, whilst about 40% disagreed. About 40% felt that either with or without HIV, they would not be happy to deal with gay people, although an equal percentage disagreed. Only the background factor of gender correlated with homophobic level (*p* = .05, *β* = −.67).

## Discussion

The knowledge level of the Russian nursing students was equal to that measured in a Lithuanian group of students, using the same instrument in large coastal cities. In the Lithuanian group the mean score was 19.1 [10], whereas in the Russian group the mean score was 19.8 (with a theoretical maximum of 33). By contrast, previous studies using the same instrument have reported the mean scores ranging from 21.8 to 25.5 among groups of nursing student in Finland [10,18], in Germany [8], in England [9], and in Estonia [10].

There was congruence in answers given to statements in the international studies. We found a high proportion of incorrect answers in Russia, Finland, Estonia, Lithuania, Germany and Great Britain, in some of the areas of knowledge of the diseases associated with AIDS. Only 46% of the Russian students knew that opportunistic infections were commonly associated with AIDS. In Finland the percentage was 56% and in Estonia 40%, but in Germany the score reached 89%. HIV was known to be a common fatal disease in developing countries by 37% of Russian students, 36% of Lithuanian, 30% of Finnish and 15% of Estonian nursing students. Half of the Russian students knew that there is no cure for AIDS, but in Lithuania the percentage was 28. In other Baltic Sea area countries e.g. in Finland and Estonia the majority responded correctly.

About 70% of the Russian students knew that HIV cannot be transmitted by casual contact. Although about 20% responded “don’t know” and 14% “possibly”, we need to emphasize this point, because if nursing students think that a person can contract HIV by casual contact, for example in nursing care, then students could fear contracting HIV/AIDS whilst carrying out their daily nursing activities. Previous studies have shown that 89% of Finnish, 88% of German, about 88% of British and Estonian, and only 63% of Lithuanian nursing students responded correctly to this question. Another interesting thing was that only 62% of the group of Russian students knew that condoms do not give a 100% protection against HIV (correct answers: UK 81%, Germany 64%, Finland 59%, Estonia 72%, Lithuania 54%) [8-10,18], and this type of general knowledge of HIV and AIDS is extremely important. As such, studies such as these serve to highlight basic educational elements that may be overlooked or otherwise presumed.

Russian students were quite well aware of HIV and AIDS-related precautions, but there were also knowledge gaps. It is very important to know when a patient needs to be isolated, for example. If nurses are knowledgeable about the correct procedures, then HIV and AIDS-related nursing is safe and patients do not need to be unnecessarily isolated. However, only 65% of the group of Russian students knew that the degree of isolation required for patients depended on the specific infection present. The knowledge level was also low among many other groups of European nursing students [8-10,18], as have knowledge levels concerning other areas such as HIV and pregnancy [8,10]. Knowledge gaps such as these could imply that nursing education does not include enough information about nursing precautions. It is therefore essential to teach those aspects which relate to daily nursing activities, especially in countries such as Russia, where the HIV and AIDS situation is escalating alarmingly.

In this study, the nursing students’ general attitudes toward PLWHA were quite negative and many of the students expressed homophobic attitudes. The mean score for nursing students’ general attitudes was 2.75. This is lower than reported in previous studies using the same instrument in other Baltic sea countries, where the mean attitude scores have ranged from 2.81 (in Lithuania) to 3.91 (in Finland) [9,10,18]. The homophobic level in the data was 3.3, and previous studies have reported levels from 3.31 (Lithuania) to 4.65 (Finland), where the 5-point scale was worded so that 1 represents the most homophobic attitude and 5 the least homophobic attitude [9,10,18]. Previous studies have reported that the nursing students’ attitude level is correlated with their willingness to provide care to PLWHA [9,18], and knowledge level (e.g. [8]). This study showed that the group of Russian nursing students involved in the study had a moderate knowledge level and quite negative attitudes toward PLWHA. These negative attitudes and lower levels of knowledge may directly influence daily nursing activities with PLWHA. Given that the number of PLWHA in Russia is high, it is therefore crucial to ensure that nursing education includes sufficient information about this group.

The study showed that almost 80% of the students felt that they should have the right to refuse to deal with persons with HIV or AIDS, and that about 40% would refuse to care for PLWHA. It should be noted that these questions were asked hypothetically, and that agreed ethical standards of practice would guide that all patients be treated in the same way. However, such general attitudes (without a concrete caring situation) pose a challenge to nursing education. For example, Mockiene et al. [25] found that an education intervention, combined with written materials, increased nurses’ knowledge level and positive attitudes. Therefore incorporating HIV and AIDS-related modules into the nursing curriculum may have a positive effect in this matter.

PLWHA have experienced stigma in nursing care (e.g. [26]), and homophobia is one aspect of this. 72% of the group of Russian final year nursing students knew that not all homosexuals have HIV, but it still remains that one in four (28%) of Russian nursing students and one in five (20%) Lithuanian nursing students either did not know or thought that all homosexuals have HIV [8-10,18]. In this study, the background factor of gender correlated with the level of homophobia, and as in previous studies, men were seen to be a little more homophobic than women.

The study also showed that nursing students’ attitudes were more negative when the hypothetical PLWHA would be either intravenous drug users or prostitutes. They also felt more sympathy towards people who acquired HIV through blood transfusion, rather than through the use of IV drugs (agree = 89%, undecided = 5%, disagree = 6%). Earlier studies have also reported similar differences (e.g. [8,27,28]).

## Conclusions

In Russia, where the HIV infection level is very high and continuing to grow, it is crucial to educate nurses and nursing students about HIV and AIDS, especially when nursing students’ attitudes are seen to be as negative as presented in this study. This is especially poignant as these students were final year nursing students, near graduation and to commencing their nursing career. Nurses are in the front line in the prevention of HIV, and in caring for these patients and risk groups. Thus, enhancing nursing students’ knowledge of HIV and AIDS and fostering positive attitudes toward patients with HIV could only improve the quality of care for PLWHA.

In order to provide evidence-based education and nursing care, we should have a more accurate perspective of the knowledge and attitude level of nursing students. This study presents a real and valuable step in examining nursing studies in Russia, and furthermore, in a city where HIV-infection is highly concentrated. It is however, not without acknowledged limitations. Due to advancing technological and pharmaceutical developments, the instrument is now somewhat dated, so future studies should be carried out with updated instruments, e.g. by including more items related to currently available medication regimes. Additionally, the sample size of this study was quite small and purposive sampling was used, so further studies are required in order to be able to generalize the results. However, this article has highlighted items relating to the knowledge level that graduating nursing students should have when they enter nursing practice and revealed some significant shortcomings. Nursing students should also be aware of their own attitudes, because they have been shown to have an impact on the way they are working. However, this study may be seen as a starting point in investigating the situation, in order to further develop nursing education and care in a country where nursing research is only just emerging as a professional practice.
